# Severe Feeding Difficulties and Undernutrition Revealing F-Box and Leucine-Rich Repeat Protein 4 (FBXL4)-Related Mitochondrial Disease: A Case Report

**DOI:** 10.7759/cureus.117671

**Published:** 2026-10-09

**Authors:** Mouna Sabib, Omar Askander, Toufik Meskini, Said Ettair

**Affiliations:** 1 Department of Pediatric Gastroenterology, Hepatology and Nutrition, Children's Hospital of Rabat, Ibn Sina University Hospital Center, Faculty of Medicine and Pharmacy of Rabat, Mohammed V University, Rabat, MAR; 2 Department of Medical Genetics, Mohammed VI University of Sciences and Health, Rabat, MAR

**Keywords:** developmental delay, fbxl4, feeding difficulties, mitochondrial disease, undernutrition, white matter abnormalities

## Abstract

F-box and leucine-rich repeat protein 4 (FBXL4) deficiency is a rare mitochondrial disorder typically characterized by lactic acidosis, hypotonia, and developmental delay. Feeding difficulties and poor growth may dominate the presentation and initially suggest a gastrointestinal disorder.

A Moroccan girl born to consanguineous parents had reduced oral intake, recurrent vomiting, and poor growth from birth, accompanied by hypotonia and developmental delay. At eight months, she was admitted with metabolic decompensation and severe undernutrition: weight was 4.8 kg, length 60 cm, weight-for-length z score −2.30, and mid-upper arm circumference 10.5 cm, meeting the criterion for severe acute malnutrition. Blood lactate ranged from 6.35 to 15.88 mmol/L. Brain magnetic resonance imaging showed cerebral atrophy and bilateral symmetric white-matter abnormalities. Proband-only exome sequencing identified the homozygous FBXL4 frameshift variant NM_001278716.2:c.851del, p.(Pro284LeufsTer7), classified as pathogenic by the laboratory. Parental segregation analysis had not been performed. Continuous nasogastric feeding with progressively increased energy intake was associated with a weight increase to 5.8 kg over a two-month hospitalization. Despite temporary stabilization, the patient died at 12 months during a respiratory infection complicated by metabolic decompensation.

This observation documents the substantial nutritional burden of FBXL4-related disease. Severe feeding difficulties accompanied by hypotonia, developmental delay, and metabolic abnormalities warrant investigation for a multisystem disorder, while nutritional support remains an immediate management priority.

## Introduction

Mitochondrial DNA (mtDNA) depletion syndromes are genetically heterogeneous disorders caused by defects in nuclear genes required for mtDNA maintenance. Biallelic pathogenic variants in FBXL4 cause encephalomyopathic mitochondrial DNA depletion syndrome 13 (MTDPS13; OMIM 615471). The disorder usually begins in the neonatal period or early infancy and is characterised by persistent lactic acidosis, hypotonia, global developmental delay, feeding difficulties and poor growth [1-5].

Involvement may be multisystemic, including neurological, cardiac, hepatic, ophthalmological, auditory and haematological manifestations [3-5]. When vomiting, feeding difficulties and undernutrition dominate the presentation, a primary gastrointestinal disorder may initially be suspected, delaying the recognition of mitochondrial disease. Coexisting hypotonia, developmental delay, persistent hyperlactataemia or symmetric brain abnormalities should therefore raise suspicion of a mitochondrial disorder.

We report a girl with the homozygous FBXL4 variant NM_001278716.2:c.851del, p.(Pro284LeufsTer7). Our objective was to document an infantile course dominated by severe feeding impairment and undernutrition, identify the findings that led to diagnosis, and compare this course with the previously published neonatal presentation associated with the same predicted protein consequence [6].

This case was previously presented as poster PA-22 at the Spring-Summer Meeting of the Société Française pour l’Étude des Erreurs Innées du Métabolisme (SFEIM), held in Strasbourg, France, from June 30 to July 1, 2026 [7].

## Case presentation

The patient was a girl born to consanguineous Moroccan parents. The pregnancy was complicated by diet-controlled gestational diabetes. Delivery was by elective cesarean section at 39 weeks of gestation. Birth weight was 1,500 g, consistent with severe intrauterine growth restriction. Neonatal adaptation was reportedly satisfactory. However, congenital ventricular enlargement required neonatal hospitalization and neuroradiological follow-up. Her two older siblings were healthy, and no similar family history was reported.

Reduced oral intake and recurrent postprandial vomiting were present from birth, accompanied by poor growth, hypotonia, and developmental delay. Head control was achieved at six months, but independent sitting had not been acquired. Worsening feeding refusal prompted admission at eight months. Clinical feeding assessment identified weak sucking and swallowing difficulties with episodes suggestive of aspiration.

On admission, weight was 4.8 kg and recumbent length was 60 cm, yielding a weight-for-length z-score of −2.30 according to the World Health Organization (WHO) Child Growth Standards, consistent with moderate wasting [8]. However, the mid-upper arm circumference of 10.5 cm met the WHO criterion for severe acute malnutrition in children aged 6-59 months (<11.5 cm) [9]. Head circumference was 40.5 cm. She was alert and responsive but pale and dehydrated, with axial hypotonia. Examination also revealed nystagmus, tachypnea without signs of increased work of breathing, and a liver edge palpable 2 cm below the right costal margin.

Laboratory investigations revealed metabolic acidosis, intermittent hypoglycemia, and marked hyperlactatemia, accompanied by anemia and transient thrombocytosis. Selected biochemical and hematological findings and reference intervals are summarized in Table 1. Upper gastrointestinal endoscopy showed no abnormalities.

**Table 1:** Biochemical and hematological findings during the clinical course

Parameter (unit)	Patient result	Reference interval
Bicarbonate / total CO₂ (mmol/L)	<10-19	18-27
Glucose (mmol/L)	2.05 (nadir)	3.6-7.7
Lactate (mmol/L)	6.35-15.88	0.90-1.70
Ammonia (µmol/L)	52.4	11-51
Aspartate aminotransferase (U/L)	66	0-110
Alanine aminotransferase (U/L)	49	0-28
Creatine kinase (U/L)	71	50-263
Hemoglobin (g/L)	72	113-141
Platelet count (× 10⁹/L)	1,000	150-450
Urea (g/L)	0.72	0.09-0.30
Creatinine (mg/L)	2.6	2.0-4.0
Prothrombin activity (%)	98	70-130

Brain magnetic resonance imaging (MRI) showed ex vacuo ventriculomegaly, thinning of the corpus callosum, widened cortical sulci, and bilateral symmetric deep and subcortical white-matter abnormalities, including involvement of the U-fibers. The lesions were hyperintense on T2-weighted and fluid-attenuated inversion recovery sequences, hypointense on T1-weighted sequences, and hyperintense on diffusion-weighted images (Figure 1). The cerebellum and brainstem were preserved. Magnetic resonance spectroscopy showed myo-inositol and choline peaks; no lactate peak was reported. Echocardiography identified a 2-mm patent ductus arteriosus with a left-to-right shunt, without hemodynamic consequences or cardiomyopathy.

**Figure 1:**
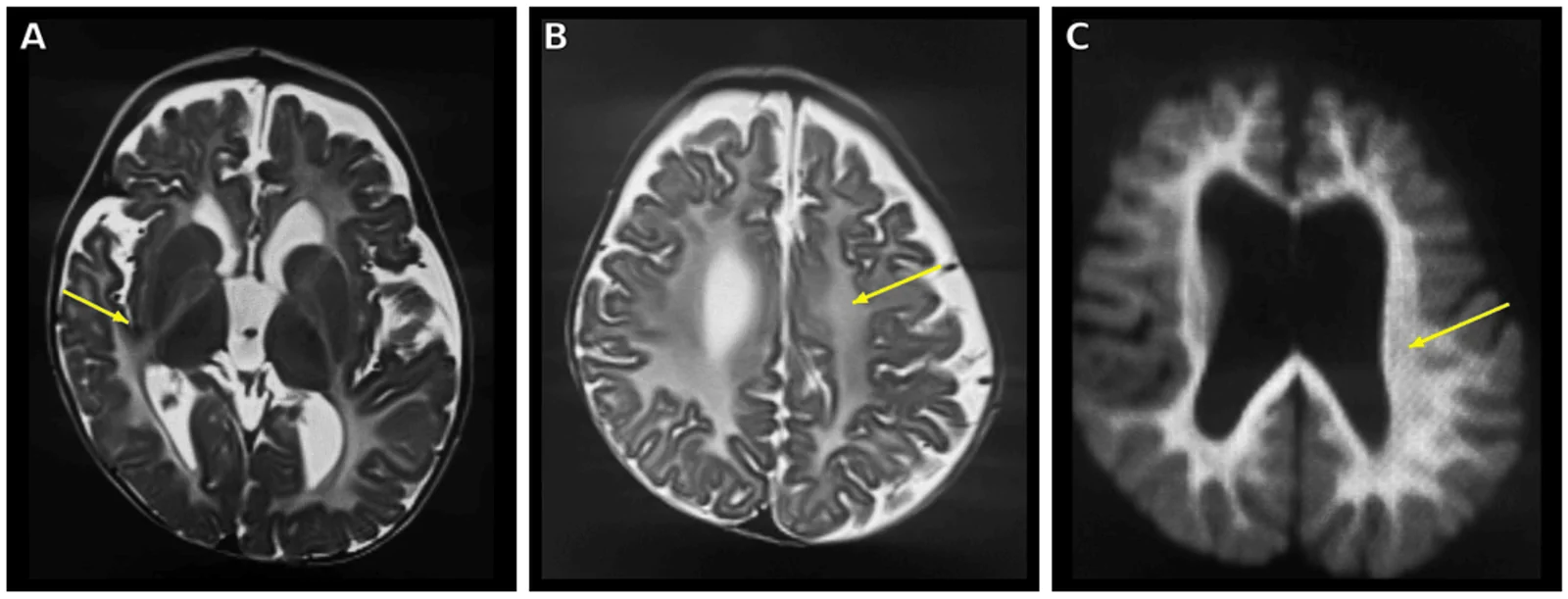
Brain magnetic resonance imaging. Yellow arrows indicate bilateral deep and subcortical white-matter hyperintensity on axial T2-weighted images (A, B) and periventricular white-matter hyperintensity on diffusion-weighted imaging (C). Ventricular enlargement and widened cortical sulci are also visible. These findings support cerebral involvement but are not specific to FBXL4-related disease.

Plasma amino acid analysis showed mildly reduced glycine, ornithine, and aspartic acid concentrations, without a specific diagnostic pattern. Urinary organic acid analysis revealed substantial lactate excretion, marked ketosis, increased tricarboxylic acid cycle intermediates and hydroxylated dicarboxylic acid derivatives, and the presence of 3-methylglutaconic and 3-methylglutaric acids. Although nonspecific and potentially influenced by catabolism, these findings, together with the neurological and imaging abnormalities, raised suspicion of mitochondrial disease.

Proband-only exome sequencing was therefore performed using DNA extracted from peripheral blood. It identified a homozygous frameshift variant in FBXL4: NM_001278716.2:c.851del; NP_001265645.1:p.(Pro284LeufsTer7). The laboratory classified the variant as pathogenic and reported its absence from gnomAD v4.1. Its predicted consequence was nonsense-mediated mRNA decay or protein truncation. No orthogonal confirmation was performed because the laboratory considered the variant call to be of high quality. Parental segregation analysis and tissue mitochondrial DNA quantification were not performed. Functional studies of the mitochondrial respiratory chain could not be undertaken because of limited resources. The diagnosis of FBXL4-related disease was based on the combined clinical, biochemical, imaging, and molecular findings.

Management included intravenous glucose to limit catabolism and correction of fluid, electrolyte, and acid-base disturbances. Feeding difficulties required exclusive nasogastric nutrition, delivered continuously over 24 hours using a hydrolyzed cow’s milk protein formula concentrated to 1 kcal/mL. Energy intake was progressively increased from 60 to 180 kcal/kg/day over 14 days, with protein intake gradually increased to 3 g/kg/day. Thiamine, phosphate, and trace elements were administered during nutritional rehabilitation. Biotin, coenzyme Q10, and carnitine were also given empirically.

A ketogenic diet was briefly attempted empirically for suspected mitochondrial energy dysfunction but was discontinued because of vomiting, somnolence, worsening acidosis, and hypoglycemia.

Progressive nasogastric feeding was clinically well tolerated, with no reported clinical manifestations of refeeding syndrome. Weight increased from 4.8 to 5.8 kg during the two-month hospitalization, with temporary clinical stabilization. Gastrostomy was considered because of persistent feeding difficulties.

After discharge, the patient died at 12 months during a respiratory infection complicated by severe metabolic decompensation. The clinical course from birth to death is summarized in Figure 2.

**Figure 2:**
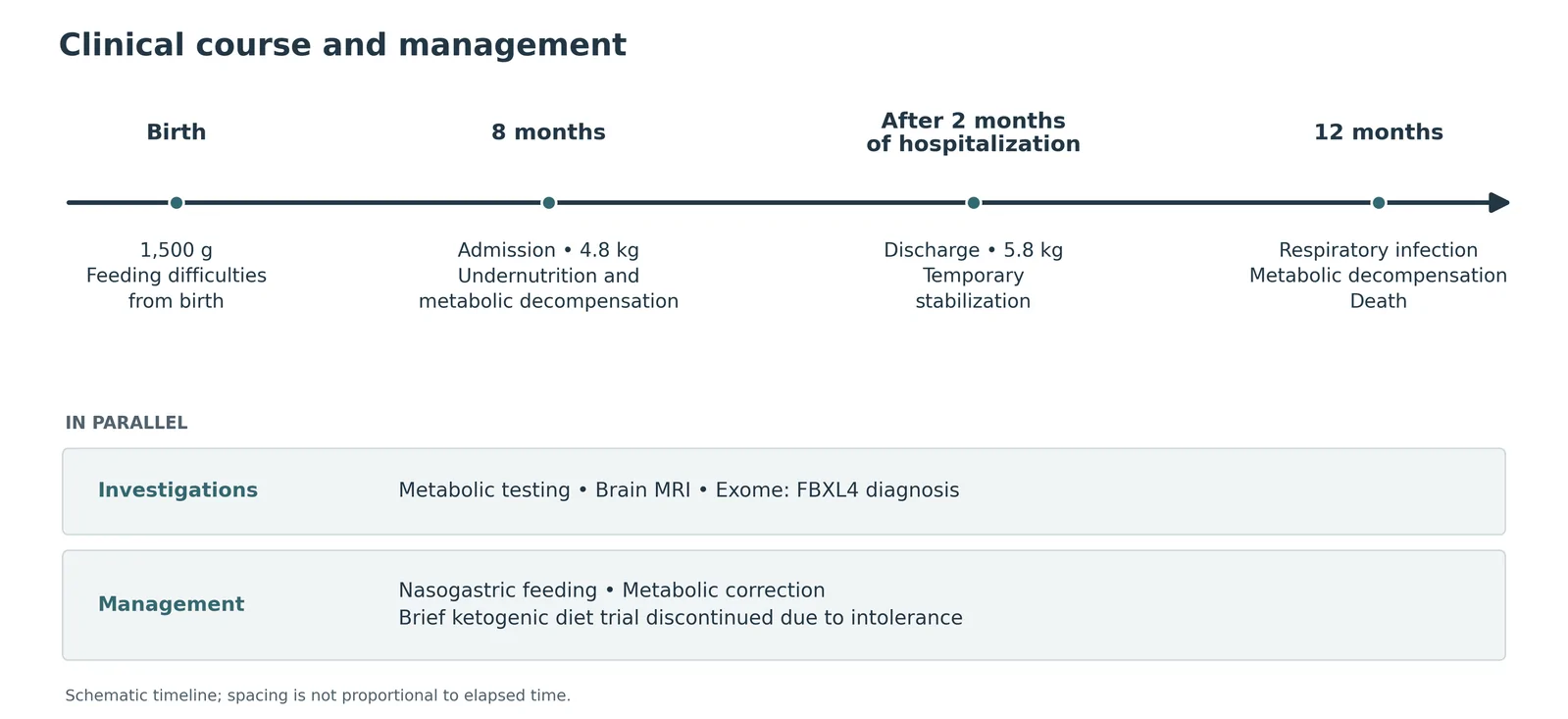
Clinical timeline and management. Diagnostic investigations proceeded alongside supportive care. Clinical timeline and management, based on the patient’s medical records, with assistance from ChatGPT (OpenAI, San Francisco, CA) for graphical layout and verified by the authors. Timeline not to scale. MRI: magnetic resonance imaging.

## Discussion

Feeding difficulties and poor growth are recognized features of FBXL4-related mitochondrial disease [3-5]. In our patient, reduced oral intake and recurrent vomiting were present from birth, and severe undernutrition became a major reason for hospitalization. The clinical interest of this observation lies in the prominence and nutritional consequences of these manifestations. Associated hypotonia, developmental delay, and metabolic abnormalities indicated a multisystem disorder and prompted investigation beyond an isolated gastrointestinal condition.

Nutritional assessment identified moderate wasting according to the WHO weight-for-length z-score and severe acute malnutrition according to the mid-upper arm circumference criterion [8,9]. This distinction clarifies the basis for the severity classification. Continuous nasogastric feeding enabled progressive delivery of energy and protein, and weight increased from 4.8 to 5.8 kg during the two-month hospitalization. This documented weight gain occurred during multidisciplinary supportive care and does not establish complete nutritional recovery or the efficacy of an individual intervention. Feeding and swallowing were assessed clinically, without instrumental evaluation, limiting characterization of the swallowing disorder and confirmation of suspected aspiration.

Emperador et al. described a boy of Moroccan origin carrying a homozygous FBXL4 frameshift variant with the same predicted protein consequence, p.(Pro284LeufsTer7) [6]. He developed neurological and respiratory deterioration on day three, with severe hyperlactatemia, hyperammonemia, and pulmonary hypertension, and died on day four. Our patient was also symptomatic from birth, but her course was dominated by persistent feeding difficulties and undernutrition, with survival to 12 months. The clinical comparison is summarized in Table 2. These contrasting courses are consistent with phenotypic heterogeneity despite the same predicted protein consequence. Genetic background, environmental factors, metabolic stress, and differences in clinical management may contribute to this variability, although their respective roles cannot be determined from these two observations. This comparison does not establish a variant-specific prognosis.

**Table 2:** Selected clinical features of the present patient and patient S2 reported by Emperador et al. [6] MRI: magnetic resonance imaging.

Feature	Present patient	Emperador et al. [6], patient S2
Variant nomenclature	NM_001278716.2:c.851del; p.(Pro284LeufsTer7); homozygous	NM_012160.4:c.851delC; p.Pro284LeufsTer7; homozygous
Parental segregation	Not yet performed	Heterozygosity confirmed in both parents
Onset and predominant presentation	Symptoms from birth; severe feeding impairment, undernutrition, vomiting, hypotonia and psychomotor delay	Acute neurological and respiratory deterioration on day 3
Maximum lactate	15.88 mmol/L	25 mmol/L
Ammonia	52.4 µmol/L	224 µmol/L
Neuroimaging	Ex vacuo ventriculomegaly, thin corpus callosum, cortical atrophy and bilateral symmetric white-matter abnormalities	Ultrasound: suspected retrocerebellar haemorrhage and enlarged cisterna magna; no MRI
Outcome	Death at 12 months during a respiratory infection complicated by metabolic decompensation	Death at four days

Persistent hyperlactatemia and urinary organic acid abnormalities supported suspicion of mitochondrial disease but were not specific to FBXL4 deficiency or respiratory chain dysfunction. Fasting, catabolism, and metabolic decompensation may influence these findings. Biochemical heterogeneity has been described in FBXL4-related disease [10], including presentations resembling organic acidemia or featuring marked hyperammonemia [11,12]. In our patient, metabolic results were interpreted alongside hypotonia, developmental delay, and symmetric cerebral abnormalities. The diagnosis rested on the combined clinical and molecular findings rather than on the urinary organic acid profile alone.

The cerebral atrophy, ventriculomegaly, thin corpus callosum, and bilateral symmetric white-matter abnormalities were consistent with the reported neuroradiological spectrum of FBXL4-related disease [3-5,12]. Diffusion-weighted hyperintensity was observed, but restricted diffusion could not be confirmed because apparent diffusion coefficient maps were unavailable. No lactate peak was reported on magnetic resonance spectroscopy despite elevated blood lactate. The small patent ductus arteriosus may have been incidental; nevertheless, cardiac assessment remains relevant because congenital heart disease, cardiomyopathy, arrhythmias, and pulmonary hypertension have been reported in affected patients [4,6,12,13].

Proband-only exome sequencing identified a homozygous frameshift variant classified as pathogenic by the reporting laboratory. Nonsense-mediated messenger RNA decay or protein truncation represents a predicted consequence, rather than a mechanism demonstrated in this patient. Emperador et al. reported muscle mitochondrial DNA depletion and functional rescue in cells from their patient [6]. These results provide relevant published evidence but do not constitute functional confirmation in our patient. Parental segregation analysis, tissue mitochondrial DNA quantification, and patient-specific functional studies were not performed.

Management remains supportive, with prevention of catabolism, correction of metabolic disturbances, individualized nutritional support, and surveillance of multisystem involvement [4,5,14]. Thiamine and micronutrient supplementation accompanied nutritional rehabilitation, while biotin, coenzyme Q10, and carnitine were administered empirically. Evidence supporting the clinical benefit of mitochondrial cofactor combinations remains limited. The brief ketogenic trial was empirical and should not be interpreted as an established treatment for FBXL4 deficiency. Temporary clinical stabilization occurred during combined supportive care and cannot be attributed to a specific supplement or dietary intervention. The sustained need for enteral feeding emphasizes the importance of nutritional support throughout the course of FBXL4-related disease.

## Conclusions

This case documents severe feeding impairment as a major early clinical and management problem in an infant with FBXL4-related mitochondrial disease. The coexistence of hypotonia, developmental delay, and metabolic abnormalities supported investigation beyond an isolated gastrointestinal disorder. The reported protein consequence had previously been associated with a rapidly fatal neonatal presentation; our patient adds a descriptive example of a more prolonged, but still fatal, infantile course. Clinical findings and nutritional needs should guide assessment and support, while prognostic interpretation should remain cautious.
